# Small peptides play a key role as messengers in plant growth, development and abiotic stress resistance

**DOI:** 10.3389/fpls.2026.1857506

**Published:** 2026-08-13

**Authors:** Lingjun Lai, Yanling Zhang, Zengting Chen, Guocheng Ren

**Affiliations:** 1 Shandong Provincial Key Laboratory of Plant Stress, College of Life Sciences, Shandong Normal University, Jinan, China; 2 Dongying Key Laboratory of Salt Tolerance Mechanism and Application of Halophytes, Dongying Institute, Shandong Normal University, Dongying, China

**Keywords:** abiotic stress, plant growth and development, regulatory mechanisms, signal molecules, small peptides

## Abstract

Against the backdrop of global environmental change, plant growth and development face multiple challenges, and a range of abiotic stresses seriously affect their normal growth. In recent years, plant small peptides (SPs)-widely defined as peptides of fewer than 100 amino acids-have emerged as important signaling molecules at the forefront of stress response strategies, playing a key role in regulating plant growth, development, and stress adaptation. This paper reviews the classification of small peptides and their effects on plant growth and development, with a particular focus on the dynamic changes and regulatory mechanisms of various small peptide families under abiotic stress. Studies have shown that small peptides regulate root development, promote cell division and growth, and control reproductive development by participating in intercellular communication, hormone signaling, and stress response networks. The aim of the review is to summarize recent advances in the understanding of SPs in plant growth and development as well as in responses to abiotic stress. We also discuss future research directions concerning the application of these molecules in crop improvement and the enhancement of environmental resilience, thereby providing a theoretical foundation for the elucidation of stress tolerance mechanisms and the breeding of stress-resistant crop varieties.

## Introduction

The growth and development of plants face multiple challenges amid intensifying global environmental change. This process is affected by both internal and external factors. Among these, abiotic stresses-such as drought, extreme temperature, salt damage, and heavy metal stress-seriously impair normal plant development. Therefore, plants have evolved a series of strategies to cope with stress in their morphological structure and physiological functions (Chen et al., 2024a). Plant small peptides (SPs) broadly refer to short proteins composed of fewer than 100 amino acids, encompassing a range of well-recognized classes, including small signaling peptides, secreted peptides, and peptide hormones. As key signaling molecules or hormones, SPs play regulatory roles in plant development and environmental responses, including growth and development as well as abiotic stress responses (Hsu and Benfey, 2018).

The study of small peptides originated with the discovery of insulin, an animal small peptide, in the early Twentieth century, and this discovery was awarded the Nobel Prize in Physiology or Medicine in 1923. It was not until 1991 that the first functional small peptide in plants, systemin, was reported in tomato. This endogenous polypeptide, composed of 18 amino acids and isolated from tomato leaves, regulates the plant’s response to pests and diseases by initiating signal transduction (Pearce et al., 1991). Since then, new SPs have been discovered, and their functions in plant growth, development, and stress responses have been progressively elucidated. These small peptides interact with specific receptors to build a sophisticated defense network, forming peptide-receptor complexes that activate downstream signaling pathways and help plants adapt to various abiotic stress conditions.

Although the functions of small peptides in plants have been reviewed previously, the rapid advancement of related fields has driven substantial progress in small peptide research, making an updated synthesis urgently needed. Therefore, this review aims to present recent findings on small peptides and summarize current advances in their roles and regulatory mechanisms under abiotic stress in plants. This review is structured around different small peptide families, with a focus on their regulatory mechanisms under abiotic stress and the key biological processes they modulate. This review summarizes recent findings on small peptides in plant abiotic stress responses and provides a reference for improving agricultural productivity.

## Classification of plant small peptides

Since the first small peptide was reported, thousands of SPs and dozens of peptide families have been identified through bioinformatic analysis in recent years. As key signaling molecules in plant physiological processes, they respond to various stress signals via distinct signaling pathways, such as those involved in ion homeostasis, ROS bursts, and hormonal signaling. Despite their wide diversity and involvement in distinct signaling networks, SPs exhibit remarkably conserved homologous gene structures and domains across plant species, and conform to a unified definition and normative criteria.

### Classification according to origin

Plant small peptides can be classified into two categories based on their biogenesis: precursor-derived and non-precursor-derived peptides.

Precursor-derived peptides are generally generated from small peptide segments encoded by protein-coding genes, and possess a signal peptide at their N-terminus. Following removal of the N-terminal signal sequence or other amino acid fragments, the resulting peptides are ultimately processed into bioactive, mature small peptides (Datta et al., 2024). These precursor proteins can be either functional or non-functional. They exert their biological roles after being transported to specific sites within the plant. Examples include Pep1, which acts as a damage-associated molecular pattern (DAMP), and CAPE peptides, which are involved in the regulation of salt stress tolerance (Chien et al., 2015; Zhang et al., 2025a). Non-functional precursor peptides can be further divided into three categories: peptides incorporating posttranslational modifications (PTMs), cysteine-rich peptides (CRPs), and Non-cysteine-rich/non-PTM peptides (Olsson et al., 2019).

Non-precursor-derived peptides are generally not generated by cleavage of larger precursor proteins. These peptides arise from direct translation of short open reading frames (sORFs) or small ORFs, and are themselves the final active products, requiring no cleavage or additional maturation steps (Ong et al., 2022). Building on this classification, such peptides are commonly encoded by short open reading frames (sORFs) no longer than 300 nucleotides, producing polypeptides generally shorter than 100 amino acids; hence, they are often referred to as micropeptides or microproteins. Based on their genomic locations relative to mRNA features, sORFs fall into two main categories. The first category includes open reading frames located at the 5′ or 3′ ends, which exhibit distinct positional relationships with the main coding sequence (CDS). These include: upstream ORFs (uORFs) confined to the 5′ untranslated region; upstream overlapping ORFs (uoORFs) situated upstream of and overlapping the CDS in a different reading frame; internal chimeric ORFs (intORFs) fully embedded within the CDS but in an alternative reading frame; downstream overlapping ORFs (doORFs) that initiate inside the CDS and extend beyond it; and downstream ORFs (dORFs) located entirely within the 3′ untranslated region (Álvarez-Urdiola and Riechmann, 2025). Since the sORFs they encode are not independent genes but reside within the untranslated regions (UTRs) of canonical genes, this class of sORFs can regulate plant physiological functions by modulating the translational efficiency of the main open reading frame (ORF) or competing for shared target proteins (Li and Lan, 2019). The second class resides within RNA molecules traditionally regarded as “non-coding”, including long non-coding RNAs (lncRNA ORFs), circular RNAs (circRNA ORFs), and primary miRNA transcripts (pri-miRNA ORFs, also referred to as miORFs) (Álvarez-Urdiola and Riechmann, 2025). Although these sORFs are short, their encoding genes are often independent and complete loci widely distributed across plant genomes; because such genes frequently contain extended promoter regions and untranslated regions (UTRs), even very short ORFs may possess complex regulatory functions (Li and Lan, 2019). Current prediction and detection techniques for precursor-derived peptides are already relatively comprehensive. High-throughput and omics approaches have rapidly expanded into the functional validation of sORFs and their encoded short peptides (SEPs), and although still complex, significant advances are now anticipated (Kearly and Nelson, 2025).

### Classification according to the N-terminal sequences of peptide precursors

To date, based on the presence of an N-terminal signal peptide—as determined by secretory properties and protein structural features—and on the specific sites where they function, SPs can be classified into secreted and non-secreted small peptides.

Non-secreted small peptides exert their effects intracellularly, regulating cellular physiological and biochemical processes. Nevertheless, under specific conditions such as cell damage, they can be released into the extracellular space to act as damage-associated molecular patterns (DAMPs) that trigger plant defense mechanisms. Accordingly, they are classified into extracellular and intracellular non-secreted small peptides (Chen et al., 2023; Zelman and Berkowitz, 2023).

Secreted small peptides are transported to the extracellular space via the conventional protein secretion pathway, and typically function as key signaling molecules in intercellular communication. This category includes extracellular post-translationally modified secreted small peptides and extracellular cysteine-rich secreted small peptides (Chen et al., 2023). Among them, cysteine-rich peptides typically contain an even number of cysteine residues at the C-terminus, which form multiple intramolecular disulfide bonds; this compact disulfide network confers a stable three-dimensional structure, thereby conferring strong resistance in complex environments and maintaining biological activity (Olsson et al., 2019). Post-translationally modified peptides are typically derived from their precursor proteins and undergo modifications such as proline hydroxylation, hydroxyproline arabinosylation, and tyrosine sulfation (Matsubayashi, 2014). Tyrosine sulfation modification depends on the tyrosine protein sulfotransferase (TPST) enzyme. Studies have shown that TPST is involved in root meristem maintenance, phosphate deficiency responses, and Casparian strip formation. Four types of sulfated peptides have been identified in *Arabidopsis*: phytosulfokines (PSKs), plant peptide-containing sulfated tyrosines (PSYs), root meristem growth factors (RGFs), and Casparian strip integrity factors (CIFs) (He et al., 2024). They and their receptors play an important role in plant development and stress adaptation as key regulators (He et al., 2024). In addition, most secreted peptides are released into the extracellular matrix via the endoplasmic reticulum-dependent pathway and participate in regulating extracellular growth and development processes, while most of the non-secreted peptides function intracellularly and are often involved in cellular defense mechanisms to help plants respond to diverse abiotic stresses (Ren et al., 2024).

## Peptide hormones within the SPs

They differ from the six commonly identified plant hormones-auxin, gibberellin, cytokinin, abscisic acid, ethylene, and brassinosteroids- which are small-molecular metabolites. They have trace and efficient key regulatory functions at different stages of plant growth and development. They act as intercellular signaling molecules in plants. Among SPs, functional peptides that exert their effects via receptor-mediated signaling to regulate growth, development, and stress responses are defined as peptide hormones, which act at very low concentrations (Lease and Walker, 2006). In recent years, peptide hormones have been identified in various plants. Their roles in plant growth and development involve but are not limited to self-regulation, reproductive development, stem cell regulation, plant structure, tissue differentiation, organogenesis, cracking, senescence, plant-pathogen and plant-insect interactions, and stress responses (Zhang et al., 2025b).

## Role of small peptides in plant growth and development

**Figure 1:**
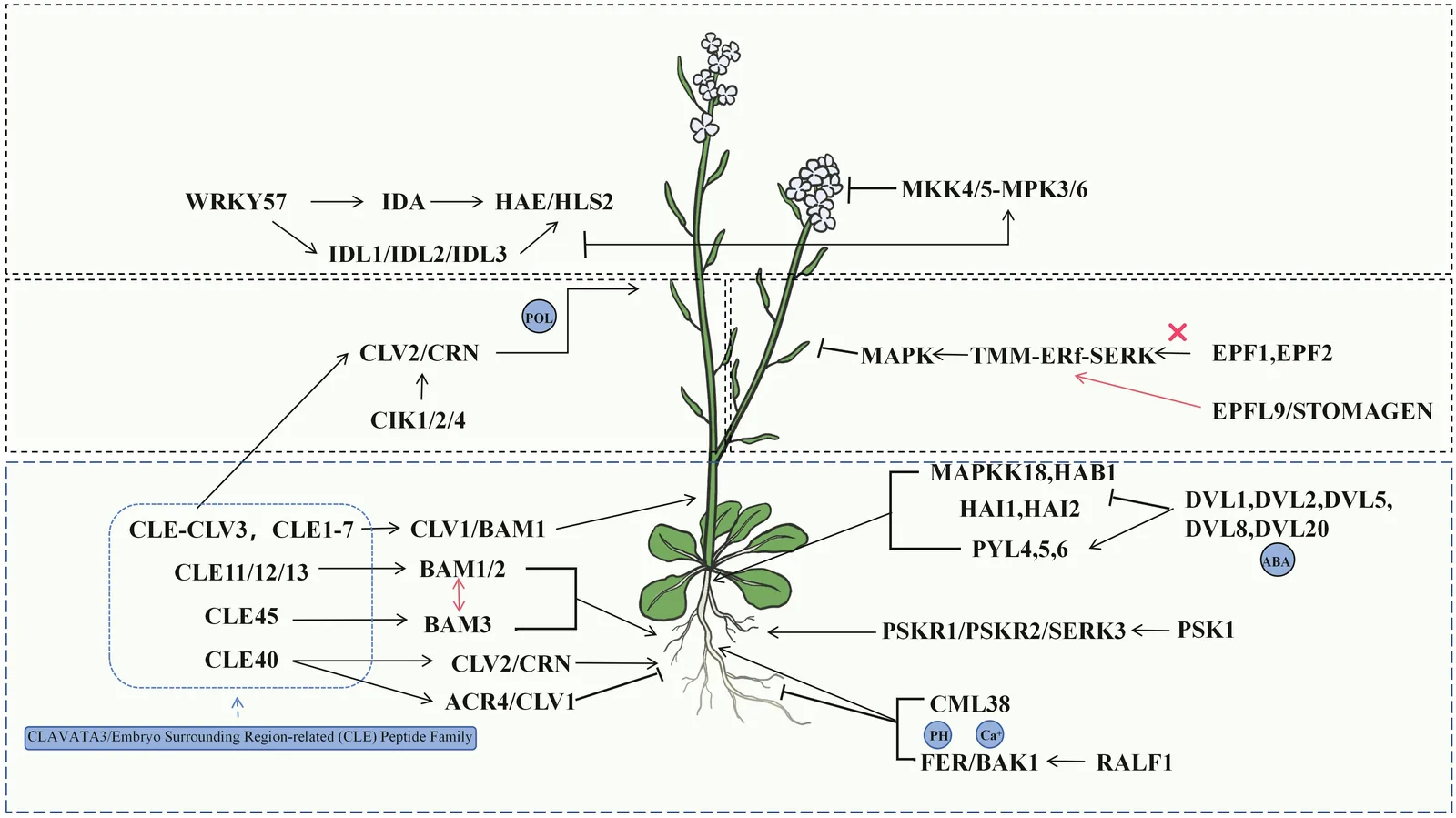
Signaling pathways of small peptides in plant growth and development. Different small peptide families play different roles in roots, stems, leaves, and flowers. CLE40, 45 small peptides, and RALF1 peptide act on different receptors to ensure the proper differentiation order of cells in the root, so that the root can continue to grow. The DVL peptide alleviates its inhibition of root development by attenuating ABA signaling. PSK1 interacts with PSKR1, PSKR2, and the co-receptor SERK3 to promote cell proliferation and lateral/primary root growth. CLE1-CLE7 peptides maintain the stability and normal development of the stem meristem through redundancy and cross-compensation mechanisms. EPF1, EPF2 and EPFL9/STOMAGEN peptides activate or compete MAPK cascades to control leaf stomatal patterns in an antagonistic manner. In addition, IDA/IDL acts on the HAE/HSL2 receptor to affect plant reproduction through the MAP cascade, while the CLV3 small peptide promotes flower growth by relying on the POL pathway. The black arrow indicates the positive regulation of the small peptide signaling pathway, the T line indicates the negative regulation, and the red arrow indicates the antagonism. The solid line represents direct adjustment, and the dotted line represents the existence of one or more unknown steps or components.

In plant growth and development, small peptides regulate key events—including stem cell maintenance, organogenesis, and stress responses—through precise intercellular communication. This section focuses on the roles of small peptides in four aspects: root development, shoot development, reproductive development, and leaf growth and stomatal development (**Table 1** and **Figure 1**).

**Table 1:** Plant small peptides related to growth and development and their functional annotation.

Peptide family	Peptide	Biological functions	References
CLE	CLE40	Promote distal root meristem cell differentiation, inhibit proximal meristem cell differentiation	(Breiden et al., 2021)
	CLE45, CLE11/12/13	Inhibit the differentiation of primary phloem sieve molecules in roots	(Endo et al., 2013; Zhang et al., 2024)
	CLE1-CLE7	Maintain the stability and normal development of the stem meristem	(Kang et al., 2022)
	CLV3/CLE	Promote flower development and ensure reproductive development of plants in thermal environments	(Jones et al., 2021; Wang and Fiers, 2010)
RALF	RALF1	Inhibition of plant lateral root development; Involved in root development	(Blackburn et al., 2020)
EPF	EPF1, EPF2, EPFL9/STOMAGEN	Participate in stomatal development, control stomatal pattern	(Lee et al., 2015; Lu et al., 2025)
PSK	PSK1	Cell proliferation and lateral and primary root growth	(Amano et al., 2007)
	PSK-ϵ	Positive regulation of root elongation and formation of lateral roots and nodules in *M.truncatula*	(Di et al., 2022)
DVL	DVL1, DVL2, DVL5, DVL8, DVL20	Reduce ABA signal transduction to regulate root and stem growth	(Yang et al., 2024)
IDA/IDL	IDA/IDL	Regulating the abscission of floral organs	(Galindo-Trigo et al., 2024; Patharkar and Walker, 2015; Stenvik et al., 2008)

### Small peptides modulate root system development in plants

Studies have shown that the signaling mechanisms of SPs are essential for root development, functioning to maintain root meristem homeostasis, inhibit differentiation, suppress lateral root formation, and regulate root hair growth.

CLAVATA3/Embryo-Surrounding Region-Related (CLE) peptides are a class of root-derived signaling peptides in plants. In the communication of the root meristem, CLE40 promotes the differentiation of distal root meristem cells through ACR4 and CLV1. Conversely, CLV2 and CRN inhibit the differentiation of proximal meristem cells, thereby promoting lateral root development (Breiden et al., 2021). CLE45 binds to BAM3, a member of the LRR-RLK receptor family, and inhibits the differentiation of primary phloem sieve elements in roots. This inhibition ensures the proper order of cell differentiation in the root, allowing the root to continue growing (Endo et al., 2013). Recent studies have shown that in *Arabidopsis* root meristems, BAM3-mediated CLE45 signaling antagonizes the initial BAM1/2-mediated CLE11/12/13 signaling in the phloem, forming this specific root meristem pattern (Zhang et al., 2024). The rapid alkalization factor RALF peptides induce rapid alkalization of extracellular compartments in plant cells. In *Arabidopsis*, the RALF1 peptide is considered a component of the root growth signaling pathway. It inhibits the development of plant lateral roots by binding to receptors FER and BAK1, and is found to interact with calmodulin-like 38 (CML38) in a Ca^2+^ and pH-dependent manner to participate in root development (Blackburn et al., 2020). Inhibition of lateral root development ensures the preferential growth of the primary root and helps plants optimize root structure under limited resources, enabling them to thrive in a variety of environments.

ROT-FOUR LIKE/DEVIL (RTFL/DVL) peptides represent a conserved class of non-secreted polypeptides. In *Arabidopsis thaliana*, this family comprises more than twenty genes encoding DVL small peptides, and numerous members are also present in crops such as *Sorghum* and *Oryza* (Wen et al., 2004). DVL1, DVL2, DVL5, DVL8, and DVL20, five members of the DVL peptide family, can alleviate the ABA-mediated suppression of root growth by attenuating ABA signaling in root stem cells (Yang et al., 2024).

For instance, PSK1 in *Arabidopsis* interacts with PSKR1, PSKR2, and the co-receptor SERK3 to form a complex that promotes cell proliferation and lateral/primary root growth (Amano et al., 2007). The precursor protein of the PSK member PSK-ϵ, designated *MtPSKϵ*, is highly expressed in *Medicago truncatula* roots, particularly in root tips and emerging lateral roots (Di et al., 2022). Studies have shown that both exogenous application of the synthetic sulfated PSK-ϵ peptide and overexpression of *MtPSKϵ* significantly promote root elongation and increase lateral root number, confirming that the PSK-ϵ peptide positively regulates root elongation as well as lateral root and nodule formation in *M. truncatula* (Di et al., 2022).

In conclusion, SPs contribute to root development through various signaling pathways that are not mutually exclusive but coordinately integrated. The convergence of CLE, PSK, and RALF signaling in lateral root development underscores the existence of cross-communication among these pathways, providing a basis for future investigations into their downstream interconnections.

### Small peptides modulate shoot development in plants

Plant small peptides are also indispensable for multiple processes during shoot growth and development. In the *Arabidopsis* stem meristem, CLE peptide ligands such as CLE1-CLE7 transmit signals through CLV1/BAM1 receptors, forming a fine-tuned regulatory network. Through redundancy and cross-compensation mechanisms, this network precisely controls stem cell behavior to promote the expression of the transcription factor WUS, thereby defining the stem cell niche and ensuring the stability and normal development of the shoot meristem (Kang et al., 2022). But it is not yet clear whether CLE1-CLE7 have distinct, overlapping, or even functionally redundant roles relative to CLV3, and the extent to which they participate in stem cell regulation under different physiological or stress conditions has not been systematically addressed.

### Small peptides modulate reproductive development in plants

IDA/IDL (IDA-like) small peptides are involved in the regulation of floral organ abscission, and WRKY transcription factors mediate the upregulation of IDA/IDL. IDA/IDL peptides bind to their receptors HAE or HAESA-LIKE2 (HSL2) and activate the mitogen-activated protein kinase (MAPK) cascade, leading to phosphorylation of downstream transcription factors (Galindo-Trigo et al., 2024; Patharkar and Walker, 2015; Stenvik et al., 2008). CLE small peptides play an important role in supporting reproductive development in plants under high-temperature stress. Among them, CLV3 is involved in stem cell niche maintenance in the shoot apical meristem during plant development. CLV3, together with certain unidentified CLE peptides, signals through the CLV2/CRN pathway independently of CLV1-related receptors. Along with the co-receptors, CIK1, CIK2, and CIK4, they promote floral growth via a POL-dependent protein phosphatase pathway (Jones et al., 2021; Wang and Fiers, 2010).

### Small peptides modulate leaf growth and stomatal development in plants

During leaf development, plant small peptides act as signaling molecules that activate cellular pathways, promote cell division in leaf primordia, guide epigenetic reprogramming for cell fate establishment, and determine the formation of distinct cell types—such as mesophyll and epidermal cells—which is crucial for normal leaf morphogenesis. SPs coordinate leaf cell expansion via wall relaxation and expansions to ensure proper morphology, and may also regulate nutrient use to sustain leaf function under deficiency.

Among the cysteine-rich peptide (CRP) family, the EPF/EPFL peptide family is another group of CRPs that has been studied in depth, second only to the RALF peptide. Several EPF/EPFL peptides have been shown to be involved in stomatal formation in *Arabidopsis* as well as in other plant species such as wheat and rice, and they are considered major regulators of stomatal development (Lu et al., 2025). Within the EPF/EPFL peptide family, EPF1 and EPF2 act as negative regulators of stomatal development, whereas EPFL9/STOMAGEN serves as a positive regulator secreted by mesophyll cells. Together, they modulate the TMM-ERF-SERK receptor complex to activate or compete with MAPK cascades, thereby antagonistically controlling stomatal patterning (Lee et al., 2015; Lu et al., 2025).

## The function of small peptide families under different abiotic stresses

### Salt stress: signal pathway activation and ion homeostasis regulation

**Figure 2:**
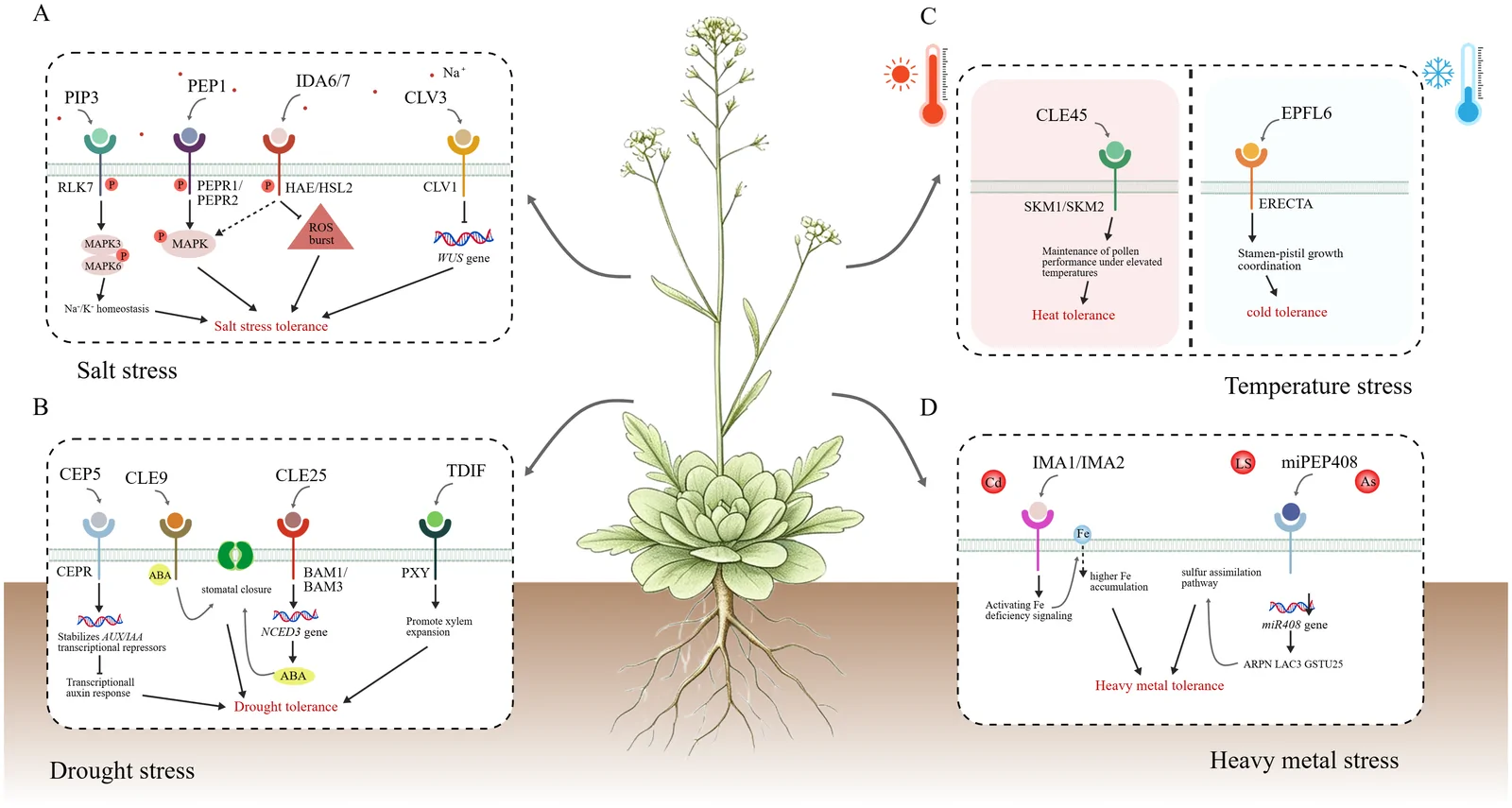
Signaling pathways of SPs under abiotic stress. **(A)** SPs participating in salt stress responses. This schematic diagram depicts, in part, the signaling cascades mediated by PIP3, PEP1, CLV3, and IDL6/7 that modulate plant responses to salt stress. **(B)** SPs involved in the regulation of drought stress. The signaling pathways mediated by CEP5 and TDIF are involved in plant responses to drought stress, whereas CLE9 and CLE25 promote drought tolerance by regulating stomatal movement. **(C)** SPs associated with temperature stress regulation. CLE45 under heat stress and EPFL6 under cold stress both serve to protect reproductive development in plants. **(D)** SPs involved in metal homeostasis. The IMA1/IMA3 peptides and the pri-miR408-encoded small peptide miPEP408 coordinate metal homeostasis under heavy metal stress (Created with https://www.BioGDP.com (Jiang et al., 2025)).

Global soil salinization is continuously intensifying and constitutes a primary cause of salt stress, which severely constrains plant growth and agricultural productivity. Under saline conditions, water deficit and excessive Na^+^ accumulation in cells impair multiple signaling cascades, manifesting as stomatal closure, ROS-induced oxidative injury, and metabolic impairment due to K^+^ depletion (Zhou et al., 2024). These detrimental effects are not uniform across all cells but ultimately inhibit plant growth by systemically disrupting intercellular communication and functional balance. SPs are regarded as critical components of signaling pathways that contribute to plant defense against salt stress (**Figure 2**).

PAMP-INDUCED SECRETED PEPTIDE 3 (PIP3) is a secreted peptide in *Arabidopsis thaliana* that is significantly induced by salt stress. Under salt stress, PIP3 is secreted into the extracellular space and interacts with the extracellular domain of the receptor-like kinase RLK7, thereby contributing to the regulation of salt stress-mediated RLK7 activation. Further studies have revealed that MAPK3/6 lies downstream of the PIP3-RLK7 module, which is required for its activation under salt stress conditions. MAPK3/6 participate in amplifying the salt-tolerance signal mediated by the PIP3-RLK7 module and initiate downstream regulatory networks, thereby enhancing plant salt tolerance (Zhou et al., 2022). Although the PIP3-RLK7-MAPK3/6 cascade represents an important advance in understanding peptide signal transduction under salt stress, it also highlights a notable limitation in current research on small peptide-receptor interactions. Specifically, the evidence for PIP3-RLK7 interaction remains largely indirect; the molecular link between RLK7 and MAPK3/6 activation has not yet been fully established; and the potential cell-type specificity of this signaling module remains to be further refined, as current analyses rely on whole-seedling measurements. AtPep1, a representative member of the Peps family in *Arabidopsis thaliana*, is released upon salt stress and associates with the plasma membrane-localized receptors PEPR1/PEPR2. This interaction activates the downstream MAPK signaling cascade, leading to the transcriptional upregulation of multiple defense genes and ultimately strengthening plant tolerance against salinity (Raghavender and Sowdhamini, 2015). However, which specific MAPK pathway is activated downstream, and whether it converges with the same cascade triggered by the aforementioned PIP small peptides, remain to be further investigated.

CLV3 (Clavata3) is a small peptide that functions critically in cell fate determination within the plant shoot apical meristem (SAM). Under salt stress, CLV3 can regulate the number and activity of stem cells in the meristem to ensure that plants can maintain certain growth ability under adverse conditions. CLV3 interacts with its receptor CLV1 to regulate stem cell fate by inhibiting WUS (Wuschel) gene expression, thereby enabling plants to maintain stable growth under salt stress (Brand et al., 2002; Lopes et al., 2021). Studies have shown that IDA subfamily members IDL6 and IDL7 are induced by various stress treatments, act as negative regulators of genes related to early stress response in *Arabidopsis*, and play a role in the negative feedback loop of the rapid stage of salt-induced ROS (Reactive Oxygen Species) burst (Vie et al., 2017). This process may depend on HAE and HSL2, the canonical receptors of the IDA family, and thereby participate in ROS regulation. This signaling event is often accompanied by the activation of the mitogen-activated protein kinase (MAPK) cascade, leading to phosphorylation of downstream transcription factors. Further clarification of the downstream targets involved in signal transduction is critically important for deciphering the specificity of signaling pathways.

Collectively, these findings underscore the essential role of SPs in conferring stress tolerance in plants under high-salt conditions and thus emphasize the multiplicity of their regulatory mechanisms in salt stress responses. Elucidating the evolutionary conservation of these peptides holds promise for guiding the targeted genetic improvement of crops cultivated on saline–alkaline soils, and for promoting the sustainable use of marginal agricultural lands.

### Drought stress: stomatal regulation and water use optimization

Drought stress seriously affects the water balance and metabolic activities of plants. Plants reduce damage by sensing drought signals and initiating corresponding defense mechanisms. The small peptide signaling pathway plays a key role in this process (**Figure 2**).

The CEP (C-terminal peptide) family is a class of small peptides that are widely present in plants. Under drought conditions, CEP small peptides are cleaved and released outside the cell, bind to the receptor CEPR, and activate downstream signaling pathways. Studies have shown that both exogenous application and overexpression of the *Arabidopsis* CEP5 peptide protect *Arabidopsis* against osmotic stress. This protective effect may be mediated by CEP5 peptide signaling, which stabilizes AUX/IAA transcriptional repressors, thereby modulating auxin signaling (Smith et al., 2020). Recent studies have found that some CEP peptides, such as NtCEP5, NtCEP9, NtCEP14, and NtCEP17, can promote the osmotic tolerance of tobacco plants and further enhance their drought stress resistance (Pan et al., 2024). The ability of CEP small peptides to enhance drought stress tolerance in tobacco reflects the high conservation of these peptides across different plant species.

Beyond their recognized roles in plant growth and development, members of the CLE small peptide family, including CLE25 and CLE9, have been shown to mediate the dehydration stress response in *Arabidopsis thaliana* (Takahashi et al., 2018). CLE25 peptide enhances NCED3 expression through the CLE25-BAM module, thereby affecting ABA biosynthesis and inducing stomatal closure. Stomatal closure induced by CLE9 is dependent on endogenous abscisic acid (ABA) signaling and is associated with the activation of MPK3 and MPK6 pathways, a process that functions independently of the BAM/CLV1 receptors (Takahashi et al., 2018; Zhang et al., 2019). Although the cognate receptor for the CLE9 peptide remains unknown, these two seemingly parallel CLE peptides signaling pathways together provide robust evidence for the complex signaling mechanisms that plants employ to cope with drought stress.

TDIF is also a small peptide hormone in the CLE family. It is independently derived from two CLE members, CLE41 and CLE44. It interacts with PXY, a leucine-rich repeat receptor kinase (LRR-RLK), to constitute the TDIF-PXY signaling pathway. The signaling pathway is primarily expressed in plant cambial cells and regulates the development of vascular tissues. Under drought conditions, TDIF binds to PXY, promotes the differentiation and expansion of xylem, increases the ability of plant water transport tissues, and thus improves the adaptability of plants to drought stress (Etchells et al., 2016).

Collectively, these observations suggest that plants use diverse SP-dependent signaling cascades to combat drought, and that these cascades form an intricate, interactive network. However, the detailed mechanisms underlying the synergistic or antagonistic interplay among these signals await further investigation.

### Temperature stress: coordination of reproductive development protection and cell stress response

Temperature stress, including high temperature and low temperature, is one of the main environmental factors that cause serious damage to plant growth and development, particularly to reproductive development. SPs have been demonstrated to perceive temperature fluctuations at multiple levels and to initiate adaptive mechanisms accordingly (**Figure 2**).

It has been proven that high-temperature stress disrupts biological membranes, triggers a massive burst of ROS, disrupts cell structures and enzymatic activities, affects reproductive development, leads to a decrease in yield, and reduces photosynthetic efficiency by inhibiting photosynthetic genes (Kan et al., 2023; Wu et al., 2025). Although there are few studies on the resistance of small peptides to high-temperature stress, there is still a huge gap. Among CLE peptides, CLE45 is one of the most fully studied high-temperature-responsive small peptides, which is induced to express at high temperature. In addition to the negative effect of CLE45 on the differentiation of protoderm cells through BAM3, the CLE45 peptide expands its expression domain from the stigma to the transmitting tract for pollen tube elongation under high-temperature conditions (30°C). It directly and specifically binds to the SKM1/SKM2 receptor proteins to sustain reproductive development and mitigate the negative impact of heat stress on seed setting rate (Depuydt et al., 2013; Endo et al., 2013). In addition to *Arabidopsis thaliana*, the CLE45-SKM1/SKM2 signaling pathway is also expressed in rice and tomato, where it protects against mitochondrial decay in pollen tubes under high-temperature conditions, maintains normal pollen tube elongation, and thereby reduces the impact of heat stress on fertilization (Endo et al., 2013). The seed setting rate of CLE45-overexpressing plants was significantly higher than that of the wild type, revealing its key role in reproductive development. This study verified the temperature-dependent regulation mechanism of CLE45 in monocot crops for the first time (Endo et al., 2013).

Small peptides also contribute to tolerance against low-temperature stress. Low-temperature stress leads to ion imbalances, such as cytoplasmic Ca^2+^ imbalance, as well as alterations in metabolic regulation and ROS levels (Guo et al., 2022; Ming et al., 2025). Successful sexual reproduction in plants is also a physiological process sensitive to thermal stress, and secretory molecules, including signal peptides, play roles in reproductive steps. Studies have shown that at low and moderate temperatures, the secreted peptide EPFL6 in *Arabidopsis thaliana* is essential for promoting the simultaneous growth of reproductive tissues, and the EPFL6-ER signaling pathway mediates stamen-pistil growth coordination (Negoro et al., 2023). Many miRNAs have previously been shown to play important regulatory roles in response to low-temperature stress. In recent years, peptides directly translated from short open reading frames (sORFs)-namely, pri-miRNA-encoded functional small peptides (miPEPs) have also emerged as important regulators in this process (Lauressergues et al., 2015). For example, the vvi-miPEP172b and vvi-miPEP3635b peptides are highly expressed in grapevines under cold stress, and their expression improves plant cold tolerance (Chen et al., 2022).

Thus far, few small peptides have been linked to temperature stress, and their mechanisms are poorly understood, with the current repertoire insufficient for a systems-level view. Future research should therefore prioritize the investigation of small peptide-involved temperature signaling and molecular interplay, which will aid in unraveling the regulatory logic underlying plant tolerance to temperature fluctuations and support the breeding of high-yielding, climate-resilient crops.

### Heavy metal stress: toxicity relief and element balance maintenance

Heavy metal pollution has a toxic effect on plants and affects their normal growth and development. Certain small peptides have been extensively regarded in previous research as critical regulators participating in the maintenance of metal ion homeostasis under heavy metal stress (**Figure 2**).

IMA was identified early on as a central small peptide governing heavy metal stress responses, and it currently represents the most thoroughly elucidated signaling peptide in the context of heavy metal tolerance. In *Arabidopsis thaliana*, exposure to Cd, As, or Fe deficiency all prominently upregulate *IMA1* and *IMA3* (Meng et al., 2022). These peptides serve as long-distance signals moving from shoots to roots to activate Fe deficiency responses, and the subsequent increase in iron content improves plant tolerance to cadmium (Meng et al., 2022; Wang et al., 2023). The highly conserved functional characteristics of IMA small peptides across both graminaceous and non-graminaceous species, together with their trace-level application requirements and environmental friendliness, provide a promising avenue for improving crop productivity under metal toxicity stress.

MicroRNA-encoded small regulatory peptides miPEPs regulate corresponding miRNAs in plants. Recent studies have uncovered a novel mechanism by which pri-miR408-encoded small peptide miPEP408 participates in stress responses through the regulation of miR408 expression in *Arabidopsis thaliana*. Beyond its known targets Plantacyanin (ARPN) and Laccase3 (LAC3), miR408 also targets GSTU25, which encodes a glutathione S-transferase implicated in sulfur metabolism and xenobiotic detoxification (Kumar et al., 2023). MiR408-overexpressing plants were sensitive to low sulfur (LS), arsenite [As (III)], and combined stress, whereas they exhibited tolerance to these conditions. Significant differences were observed between these plant lines in the expression of genes involved in the sulfur reduction pathway, as well as in sulfate and glutathione accumulation (Kumar et al., 2023). The miR408-miPEP408 module is involved in the response to heavy metal stress and nutrient deficiency by regulating the sulfur assimilation pathway, providing new insights into the mechanism of plant stress adaptation.

Although some progress has been made in understanding how peptides such as IMA and miPEP408 participate in coordinating ion homeostasis under heavy metal stress, future research remains needed to integrate their activities into broader stress-signaling networks. Furthermore, elucidating the evolutionary conservation of these regulatory mechanisms may help uncover more universal strategies for stress tolerance.

## Application of plant small peptides as key tools for sustainable agriculture

Plant peptides have been established as natural regulators of growth and abiotic stress responses, and have emerged as promising molecular tools for sustainable agricultural practices. The application of SPs as plant biostimulants to enhance crop health and yield has emerged as an important strategy. Unlike traditional agrochemicals, which often raise environmental and health concerns, small peptides exhibit high target specificity, biodegradability, low toxicity, and minimal off-target effects, thereby substantially reducing safety risks in agricultural environments. Currently, the application of small peptides in crops is primarily achieved through exogenous application and endogenous regulation.

The exogenous application of synthetic plant small peptides via foliar spraying or root drip irrigation has been shown to effectively induce stress adaptation, regulate growth and development, and enhance crop yield. For instance, the synthetic peptides ENOD40 and CEP1, used as foliar-applied biostimulants, have been shown to activate the signaling cascade mediated by the receptor-like kinase PvSYMRK, leading to enhanced nodulation efficiency and improved productivity in legumes (Cántaro-Segura and Zúñiga-Dávila, 2025). Numerous functional peptides have already been employed in commercial agricultural production. For example, the functional peptide PY91 is used as a crop growth regulator, and the product Coveron Stim from Hello Nature utilizes SPs to enhance root development and improve crop resilience to environmental stress.

Exogenous application of small peptides offers the flexibility to rapidly enhance crop stress tolerance or short-term growth. However, given the high cost of synthesizing bioactive peptides and their susceptibility to environmental factors, research on endogenous regulation remains indispensable for practical applications. Targeted enhancement of plant peptide signaling can be achieved through gene editing or overexpression of endogenous peptide-encoding genes, enabling precise genetic refinement in crop breeding programs. Recent progress in genome-editing tools has enabled precise modulation of peptide–receptor pairs, thereby fine-tuning stress responses while maintaining normal growth and yield performance.

## Discussion

From high salinity to drought, from temperature stress to oxidative damage, small peptides play a crucial role in plant adaptation to abiotic stress. By interacting with specific receptors, small peptides activate complex signaling networks and regulate plant growth, development, and stress responses. These fine-regulation mechanisms not only enhance the adaptability of plants to adversity but also provide new ideas and strategies for improving crop yield and stress resistance through biotechnology.

Despite substantial progress in understanding the signaling functions of small peptides (SPs) under various abiotic stresses—including drought, salinity, extreme temperatures, and heavy metal toxicity—current research remains largely at the “average” level, with measurements performed on mixed cell populations. Although this approach provides valuable systemic insights at the whole-plant level, it inevitably obscures the functional heterogeneity and dynamic interactions among distinct cell types, which are critical for understanding how abiotic stress inhibits plant growth and development. In fact, stress-induced growth inhibition is not merely a consequence of general toxicity at the whole-plant level but, more fundamentally, reflects the disruption of functional balance among different cell types. For instance, in leaves, abiotic stress perturbs the coordination among photosynthetic mesophyll cells, guard cells that regulate gas exchange and transpiration, and vascular bundles that mediate long-distance water and ion transport. When this coordination is disturbed, carbon assimilation and water-use efficiency decline, ultimately manifesting as growth inhibition. Similarly, in roots, stress conditions disrupt the interactions between the cortex (involved in ion absorption and storage) and the pericycle (which harbors stem cell niches and gives rise to lateral root primordia), leading to altered root architecture and reduced nutrient acquisition. Future research should therefore focus on constructing signaling network models of SP-mediated intercellular communication across different cell types, identifying which SPs serve as “coordinators” between specific cell types, and elucidating how these coordination processes are perturbed and modulated under salt stress. Ultimately, this knowledge will lay the foundation for precision engineering of crop stress tolerance by targeting specific cell types and cell-cell communication pathways.

Plant small peptides play a key role in regulating multiple plant responses, and their mechanisms are diverse; most known small peptides, especially secretory peptides, regulate plant physiological processes through receptor-mediated signaling pathways. The core goal of screening peptide receptors is to identify and validate the receptor proteins involved in recognizing small peptides and to improve the small peptide signaling pathway. At present, the commonly used screening methods and technical routes include yeast two-hybrid verification, immunoprecipitation screening of interacting proteins, fluorescence-labeled receptor-ligand interaction screening, chimeric receptor experiments, structural biology method-assisted receptor screening, etc. In recent years, some high-throughput screening techniques and platforms have been developed. This micro, rapid, sensitive, and accurate method has become an effective tool for studying the transcriptional regulatory network of peptide signals. Nanosphere etching and nanoimprint technology have been used to detect small peptide-receptor binding in plants. Periodic gold nanopores were prepared by the nanoimprint ‘one-step’ method to realize real-time kinetic analysis of small peptide-receptor binding (Qi, 2019). With the future development of nanotechnology and multi-omics technology, a major goal will be to drive the screening of SPs receptors toward high-throughput and intelligent platforms.

In recent years, the application of various plant small peptides continues to hold broad research potential. With advances in analytical technology, the separation methods for plant small peptides have evolved significantly, from traditional chromatographic separation to new nanomaterial-assisted purification, from single-mode solid-phase extraction to multidimensional separation strategies, providing strong technical support for plant small peptide omics research. The separation and application of small peptides are widely studied, but the active sites of endogenous peptides are often modified in specific ways that are crucial for receptor recognition. The latest research uses nanopore sequencing technology to accurately identify the tyrosine sulfation modification of plant peptide hormone PSK at the single-molecule level for the first time, and to distinguish the modification status of adjacent tyrosine residues, which provides a new strategy for screening functionally modified small peptides (Chen et al., 2024b). Meanwhile, breaking through the constraints of conventional production methods is urgently needed. Synthetic biology-based biomanufacturing offers a critical solution to the economic challenges of mass-producing complex peptide drugs, which are hindered by post-translational modifications.

The small size, structural diversity, and trace-level activity of SPs also make their detection and identification particularly challenging. The identification of peptides by conventional mass spectrometry (MS) remains technically challenging, particularly for “non-canonical peptides” encoded by small open reading frames (sORFs) (Wang et al., 2020). Technological breakthroughs across multiple dimensions are needed to improve SPs discovery. Integrating the rapidly accumulating omics data through systems biology, combined with advanced AI tools and bioinformatics technologies, will provide a major boost in this regard.

The study of plant small peptides has been gradually advanced. In addition to model plants such as *Arabidopsis* and *Oryza*, SPs have been identified in various crops such as sorghum and rice. Homologous genes and structures in model organisms show high conservation across plant species in crops (Jeon et al., 2021). The application of plant peptides in crops is a hotspot in agricultural biotechnology research and is expected to address the bottleneck in traditional agriculture. Major translational challenges remain to be addressed before SPs-based strategies can be effectively applied in agriculture. These include the high cost of peptide synthesis, the instability of exogenous SPs under field conditions, and the need for efficient delivery systems.

In the future, interdisciplinary collaboration—integrating synthetic biology, systems biology, and precision agriculture—will enable a deeper understanding of SPs and the holistic elucidation of their regulatory networks. In particular, systems biology offers a complementary conceptual framework in which the plant is viewed as an integrated system comprising distinct components (cell types), and in which regulation arises from the dynamic interactions among these components rather than from the isolated activity of any single element (Pasternak and Yaroshko, 2026). This will facilitate the development of more effective strategies for improving crop stress tolerance and provide new breeding approaches and technical support for coping with global climate change.
